# A natural experiment of state-level physical activity and screen-time policy changes early childhood education (ECE) centers and child physical activity

**DOI:** 10.1186/s12889-020-08533-8

**Published:** 2020-03-24

**Authors:** Chelsea L. Kracht, E. Kipling Webster, Amanda E. Staiano

**Affiliations:** 1 Pennington Biomedical Research Center, 6400 Perkins Road, Baton Rouge, LA 70808 USA; 2 Institute of Public and Preventive Health, 1120 15th Street, CJ 2312, Augusta, GA 30912 USA

## Abstract

**Background:**

Early childhood education (ECE) centers are an important place for preschool-aged children to obtain physical activity (PA). A U.S. state government (Louisiana) recently updated requirements for licensed centers’ PA and screen-time policies, which allowed for assessment of 1) ECE center practices, environment, staff behaviors, and policies changes on child-level PA and 2) state level changes on the ECE center.

**Methods:**

ECE centers were assessed at the beginning of state licensing changes and 1-year later. The ECE centers were assessed via the Environmental Policy Assessment and Observation (EPAO) tool. The EPAO Sedentary Opportunities score, which primarily assesses television viewing time, was revised to reflect viewing non-television devices (e.g. tablets). Child-level PA was measured using accelerometry. For Aim 1, mixed models assessed ECE center changes and child PA with adjustment for demographic characteristics (fixed effects), baseline EPAO score (random effects), and clustering for center. For Aim 2, paired t-tests assessed ECE center environment differences between baseline and follow-up.

**Results:**

Nine ECE centers participated and 49 preschoolers provided complete measures at both time points. For Aim 1, increases in the EPAO revised-Sedentary Opportunities score (as in less non-television screen-time) resulted in increased child PA (*p* = 0.02). For Aim 2, ECE centers improved their EPAO Active Opportunities and Staff Behaviors score (*p* = 0.04 and *p* = 0.02 respectively).

**Conclusions:**

ECE centers improved their environment after 1-year, resulting in additional child PA. Changes in ECE centers environment, possibly through policy, can positively influence children’s PA.

## Background

Physical activity is paramount for appropriate growth and development of young children [1]. Despite the benefits, one-third of preschool age children (3-to-5 years of age) did not meet the physical activity guidelines including 3 hours of physical activity daily (of which 1 hour is moderate-to-vigorous physical activity [MVPA]), according to recent evidence in Canadian children [2]. Children are spending time viewing television, computer, and other screens, i.e. engaged in screen-time, which has been associated with excess weight in a cross-sectional analysis of 1809 preschool age children [3]. Screen-time guidelines for preschool children are less than 1 hour of screen-time daily [4], yet another cross-sectional study indicated few preschool age children (24.4%) meet these guidelines [2]. Opportunities to increase physical activity and decrease screen-time in preschool age children are necessary to meet age appropriate guidelines and ensure healthy development.

It is important to consider the entire day of a preschool age child, as it relates to both physical activity and screen-time. Eighty percent of preschool age children in the United States use a weekly non-parental care-based setting, including center-based care or an early childhood education (ECE) center [5] for either part-time or full-time care. ECE center practices, such as providing active opportunities including outdoor play time, have been positively associated with children’s MVPA in a study of nine Dutch preschools [6]. Further, the environment and staff at ECE centers have been targeted for intervention to facilitate more physical activity [7], as presence of fixed and portable play environment, and staff behaviors encouraging physical activity (such as participating in physical activity and positive statements about physical activity) accounted for 49.3% of the variability in children’s MVPA while at the ECE center in an observational study of 5 ECE centers [7]. Thus, practices, environment, and staff behavior within the ECE center are important factors in preschool age children’s physical activity.

ECE centers are also in a unique position to influence physical activity and screen-time; in many regions or municipalities, each center must follow health-related guidelines set by the government in order to obtain licensure [8]. Until 2015, the state of Louisiana did not have a requirement for physical activity or screen-time in ECE centers. Thus in late 2015, the Louisiana Department of Education released new licensing requirements whereby to remain licensed each ECE center must create and display both a written physical activity policy and procedure (stating that the center provides at least 1 hour of physical activity per day including both teacher-led and free play) and an electronic device policy (stating that the ECE center allows no more than 2 hours per day of screen-time) [9].

There is limited evidence of the effectiveness of governmental policy change on an ECE center’s own physical activity and screen-time policies and practices or on the children’s actual physical activity and screen-time behavior, as most studies evaluate these components in a cross-sectional manner [10]. The recent change in Louisiana’s policy provided a unique opportunity to evaluate a natural experiment of change in state policy on the ECE center environment and preschool children’s behavior while at the center. Aim 1 of this study was to evaluate the association among ECE center changes in practices, environment, staff behaviors, and policies, and changes in child physical activity and sedentary behavior while attending the ECE centers. Aim 2 of this study was to evaluate changes in ECE centers’ practices, environment, staff behaviors, and policy before and after implementation of the state physical activity and screen-time policy.

## Methods

This study utilized baseline and 1-year follow-up data from an observational cohort, the “Pause and Play” study, which aimed to assess state policy changes on Louisiana ECE centers before and after implementation of the regulation. The study was designed to occur in ten ECE centers for adequate power to compare differences before and after implementation based on previous research [11]. The Louisiana state licensure changes were enacted in late 2015 for implementation during the 2016–17 school year. The minimum standard was to post the state policies in the ECE center for viewing, such as on a bulletin board, but there were no requirements to update handbooks. Data collection for baseline occurred at the beginning of the implementation period (April 2016–June 2017). Researchers conducted follow-up measures approximately 1 year after each center’s respective baseline assessment (May 2017–May 2018). The Louisiana Department of Education conducts regular unannounced licensure inspections in ECE centers, with inspections no more than 1 year apart. Thus, the ECE centers were inspected at least once after baseline and before follow-up measures to maintain licensure. The full purpose of the study was not disclosed to directors or parents until after data were collected. The Pennington Biomedical Research Center Institutional Review Board approved the study. This study follows the STROBE guidelines for reporting of observational studies (Supplementary Table 1).

As a part of this study, a community-based partnership was formed to evaluate the influence of the policy changes [12]. A prior published investigation revealed that baseline measures of observed and director reported screen-time were related to baseline child physical activity [13]. This investigation focuses on the longitudinal measures of the study.

### Participants

Information on licensed ECE centers within a specific parish (county) of Louisiana were obtained from the state’s Department of Education. ECE centers were stratified by federal funding status, then randomized and contacted in order of randomization. Eligibility criteria included being a licensed ECE center in the specific parish, enrollment of at least 18 children between the ages of 3-to-4 years in their ECE center, and willing to participate. One hundred and forty six ECE centers were contacted, though 41 ECE centers did not respond, and 12 ECE centers were not licensed. Ninety three ECE centers participated in phone screen, though only fifteen ECE centers were eligible and five ECE centers did not participate due to changing their mind (*n* = 1), closed due to natural disaster (*n* = 1), or the study was filled before they finished enrolling (*n* = 3). The ECE director provided written informed consent for the ECE center to participate during a pre-observation visit. ECE directors selected one classroom within the age range (ages 3-to-4 years) for observation, and this same classroom was observed one-year later, thus different children might have been present in that particular classroom during the follow-up year. ECE centers received $200 in office and school supplies after both baseline and follow-up visits, and these supplies were not related to physical activity or screen-time.

Parents were recruited through an email or letter from the child’s ECE director or during in-person informational sessions at the ECE centers. Parents were given the opportunity to withdraw their child from participating in the classroom observation, though no parent refused participation. Parents were also invited to provide written informed consent that allowed their child to wear the accelerometer and participate in height and weight assessments. Children were eligible if they were 3–4 years of age, attended the ECE center full time (at least 6 h/day and 5 days/week), and planned to attend the same ECE center the following school year. Researchers invited children to participate in the study using developmental appropriate language and offered the children the opportunity to refuse to participate. Children received a small toy for each day if they wore the accelerometer (totaling less than one U.S. dollar per child overall). Parents completed a demographic form that included the child’s date of birth, sex, race/ethnicity; parental marital status, education level, and employment status; and household income.

### ECE center policy engagement

At baseline, the ECE director completed a survey about their participation in Louisiana Department of Education events related to state policy and licensure changes. These events included attending a roundtable discussion, viewing a recorded presentation, or attending a webinar. Policy engagement was categorized into no engagement (did not participate any event), some engagement (participated in 1 event), and high engagement (participated in 2 or more events).

### ECE center practices, environment, staff behavior, and policies

The physical activity component of the validated Environmental and Policy Assessment and Observation (EPAO) tool [14, 15] was used to measure each ECE center’s practices, environment, staff behavior, and policies related to physical activity and screen-time.

During a full day of observation of one classroom at each center, trained research specialists documented time children spent in active play, sedentary opportunities, and screen-time; environmental characteristics; and staff behavior. Trained research specialists completed over 20 h of training and obtained over ≥95% agreement during two practice observations prior to data collection. The trained research specialists EPAO domain and total scores were in complete agreement (100% intra-class correlation) for both practice observations. The current study used all eight physical activity domains of the EPAO, including: Active Opportunities, Sedentary Opportunities, Sedentary Environment, Portable Play Environment, Fixed Play Environment, Staff Behaviors, Physical Activity Training and Education, and Physical Activity Policy. Each EPAO domain assesses meeting physical activity standards on a 3 point scale (0 points when the action is not present, 1 points for meeting half the standard, and 2 points for meeting standard), these individual scores are summed and averaged, and then multiplied by 10 to create an EPAO domain score. Domains of the EPAO were calculated with a maximum possible score of 20 points per domain, with a higher score indicating a more supportive environment for physical activity. An overall score (“total EPAO score”), which was a sum of all domains (maximum score 160), was calculated. The following sections (ECE center practices, ECE Center Environment, ECE Center Staff Behavior, and ECE Center Policies) describe the EPAO domains further.

#### ECE center practices

Trained research specialists directly observed active play, television viewing time, non-television screen-time time, and total screen-time (including both television and screen use on non-television devices) during the day of ECE center observation. Active play was defined as an activity where more than half of the children were participating, and the children were engaged in MVPA. Non-television screen-time included viewing time of a computer, hand-held device, or tablet. A stopwatch and clock were used to record time and summed for the entire day.

#### ECE center environment

The ECE center Environment components assessed included EPAO scores of Active Opportunities, Sedentary Opportunities, and a revised-Sedentary Opportunities Score. Active Opportunities and Sedentary Opportunities scores are on observed active play and screen viewing time (see ECE Center Practices). Active Opportunities is meeting standards for active play minutes (60 or more minutes), teacher-led active play opportunities (any teacher-led opportunities), outdoor play opportunities (any outdoor play observed), and outdoor play minutes (120 or more minutes). Sedentary Opportunities included meeting standards for sedentary minutes (no more than 30 min sedentary at one time), viewing television (no viewing is the standard), viewing video games (no viewing), and length of viewing television (no viewing) and video games (no viewing). Due to the recent changes in technology, a revised-Sedentary Opportunities score was calculated utilizing screen-time from non-television devices (such as computers, tablets, or electronic toys) along with television. Sedentary Environment, Portable Play Environment, and Fixed Play Environment scores utilized observed equipment and indoor and outdoor areas. Sedentary Environment score was meeting standards for sedentary considerations including presence of TV (not present), computer or electronic device in the room for use by children (not present), and posters, pictures or books about physical activity displayed (present). Portable Environment score included the presence (2 points) or absence (0 points) of seven physical activity specific equipment (ball play, floor play, jumping play, parachute, push/pull toys, riding toys, and twirling equipment). Fixed Environment score included the presence (2 points) or absences (0 points) of having specific fixed environment items (basketball hoop, merry-go-round, see-saw, swinging equipment, and tunnels), outdoor play occurred, and size of outdoor (unobstructed for 2 points) and indoor play space (room for all activities given 2 points).

#### ECE center staff behavior

Staff Behaviors score from the EPAO was meeting staff behavior standards, including restricting play as punishment (not observed), participating activity (observed), and positive statements made about physical activity (3 or greater for 2 points, and 1 or 2 statements for 1 point, 0 points for no statements).

Physical Activity Training and Education score was a combination of observed (formal physical education lessons, extracurricular activities offered along with active alternatives for children) and director reported physical activity training opportunities (training in physical activity for preschool teachers and a documented physical activity curriculum). Presence of the standard was given 2 points and no points if the action was not observed or rarely observed.

#### ECE center policies

The EPAO Physical Activity Policy score was based on reviewing the ECE center’s handbook review of specific physical activity and TV viewing related policies, including policies related to active play and inactive play, supporting physical activity, TV use and TV viewing, and physical activity education. If the policy included at least two policies they were given 2 points, and 1 point if there was only one component.

### Child physical activity

An ActiGraph GT3X+ accelerometer (Walton Beach, FL) measured child physical activity. A trained research specialist placed the accelerometer on the child’s right hip and secured using an adjustable latex-free elastic waist/hip belt. Parents were asked that the children wear the accelerometer for 8 days continuously (24 h/day) and only to take off the accelerometer for water-based activities.

Researchers used accelerometer data recorded on observation day during the class start and end time was used for analysis. Continuous strings of zeros that exceeded 30 min were removed based on previous literature in this age range [16, 17]. Age appropriate cut-points were applied to determine sedentary behavior (SB; < 200 counts/15 s), light physical activity (200–419 counts/15 s), and MVPA (≥420 counts/15 s) while the child was at the ECE center [18, 19]. Total physical activity (TPA) was the sum of light physical activity and MVPA. All intensities were expressed as minutes/hours worn to account for child wear time. Children who wore the device for at least 4 hours while at the ECE center were included in analysis based on previous research [7, 20].

### Child anthropometry

Two measures of height (in in centimeters) and weight (in kilograms) were measured at the ECE center by a trained research specialist in a private setting with the child wearing light clothing and shoes removed. Height was measured to the nearest 0.1 cm on a portable stadiometer, and weight was measured to the nearest 0.1 kg via a high-precision electronic scale. A third measurement was taken if the two measurements differed by more than 0.5 units. Body mass index (BMI) percentile was calculated based on the child’s age and sex [21].

### Data analysis

Central tendencies were calculated to describe the sample. Aim 1 was conducted in the sub-group of 49 children who were in the same observed classroom at each center at both baseline and follow-up to assess within-classroom and within-child changes. For these children, mixed models were used to assess the change in ECE center components (random parameters) with change in child physical activity, including the covariates of the center’s baseline ECE center practice in minutes (for ECE center practice models) or EPAO domain score in points (for EPAO domain models); the baseline child age, sex, race, and BMI percentile (fixed parameters); and accounting for clustering within ECE center. For Aim 2, a One-Way analysis of variance (ANOVA) assessed differences in total EPAO score at baseline, follow-up, and overall change by policy engagement category (no engagement, some engagement, high engagement). Paired t-tests assessed the difference between baseline and follow-up ECE center practices, environment, staff behavior, and policies. Cohen’s d for paired t-tests was calculated for effect sizes. SAS statistical software package 9.4 performed all analyses and significance was set at the *p* < 0.05 level.

## Results

**Fig. 1:**
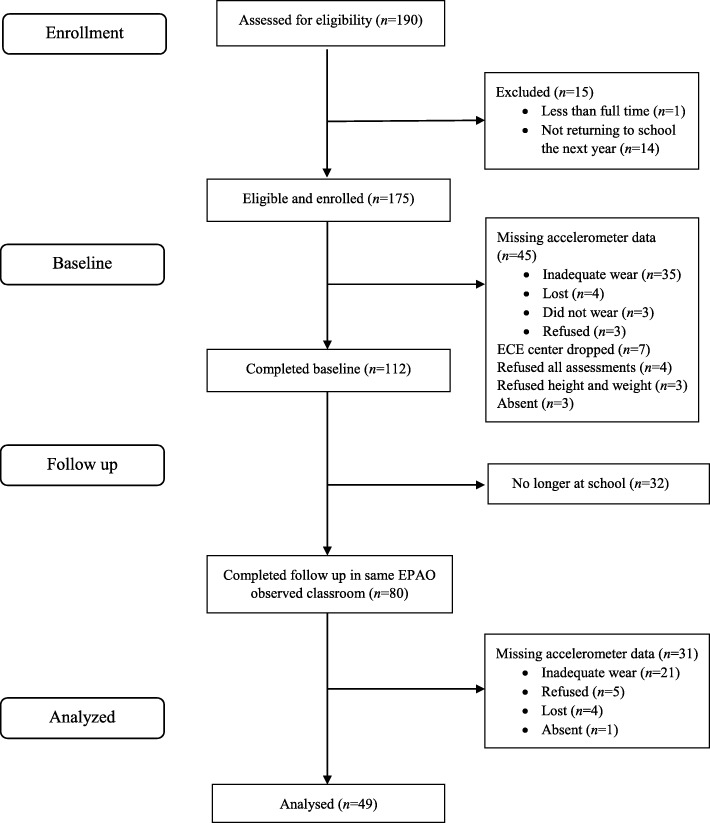
CONSORT Diagram for Participating Children

Ten ECE centers completed baseline measures, and nine ECE centers completed follow-up measures. One center that participated in baseline did not participate in follow-up measures due to small sample size (*n* = 7 children participated but *n* = 3 children had complete measures) and change in center management during follow-up year; therefore, this center could not be included in the analyses. This center enrolled fewer children ages of 3–4 years (*n* = 19) at baseline relative to other centers (range 29–194 children). One hundred and seventy-five children participated in baseline with 112 providing complete measures (64%), and 130 children participated in follow-up (including new children) with 83 children providing complete measures (63%). Forty-nine children had complete measures and were in the same observed classroom at both baseline and follow-up so were included in the Aim 1 analyses (see Fig. 1). Of the 112 children with complete measures at baseline, more children were White (57.4%) and had a higher income status compared to the other 63 children not included (32.50% White, *p* = 0.01; income status: *p* = 0.04). No other differences were found. Those who had complete measures at follow-up were slightly younger (48.1 ± 7.6 months, *n* = 83) compared to those who did not have complete measures at follow-up (51.6 ± 5.6 months, *n* = 47, *p* = 0.01) but did not differ by race, sex, or BMI percentile.

Table 1 displays the descriptive characteristics of ECE centers and children included. In total, three ECE centers were based in a church, two ECE centers were Early Head Start or Head Start Centers (ECE centers that are federally funded to provide care to low-income children), and the remaining centers were either corporate-sponsored, located in an elementary school, privately owned, or affiliated with a university. Two of the chosen classrooms included multiage children (3-to-5 years of age), while the other seven classrooms included similar aged children (ages 3-to-4 years or 4-to-5 years). Of the children who participated, most of their mothers were employed full-time (73%) or part-time (12%) at baseline and similarly at follow-up (78% full-time and 14% part-time). Researchers classified more children at follow-up as overweight (13.2%) and obese (14.2%) compared to the baseline sample (16.0% overweight and 6.2% obese), although no significant difference appeared in BMI percentile between time points.

**Table 1:** Characteristics of ECE Centers and Children included in Analytical Sample

	Baseline (*n* = 9)		Follow-up (*n* = 9)			
	Mean ± SD		Mean ± SD			
*ECE Centers*						
Number of Employees	22 ± 6		22 ± 7			
Child Enrollment						
3- year olds	43 ± 33		38 ± 27			
4- year olds	35 ± 27		30 ± 22			
	Baseline (*n* = 112)		Follow-up (*n* = 83)		Subset Baseline (*n* = 49)	
	Mean ± SD	%	Mean ± SD	%	Mean ± SD	%
*Children*						
Age	3.4 ± 0.5		4.0 ± 0.6		3.2 ± 0.5	
Boys		50.0		45.8		51.0
Race						
White		57.2		66.2		75.5
African American		33.9		25.0		14.3
Other		8.9		8.8		10.2
Ethnicity						
Hispanic		3.5		3.7		2.0
Non-Hispanic		96.5		96.3		98.0
Maternal Education						
Less than High School		2.6		2.4		2.0
High School		25.2		16.8		12.2
Associate’s/Bachelors		39.2		41.0		38.8
Graduate/Professional		33.0		39.8		47.0
Household Income (USD)						
Less than $29,999		19.1		19.5		8.2
$30,000 - $69,999		7.8		2.5		6.2
$70,000 - $109,000		14.3		13.4		12.2
Greater than $110,000		40.8		52.4		61.2
Prefer not to answer		16.0		12.2		12.2
BMI Percentile	57.2 ± 30.0		56.3 ± 32.3		52.2 ± 33.0	
Weight Status						
Underweight		6.2		6.0		10.2
Normal		71.4		66.3		67.3
Overweight		16.0		13.2		16.3
Obese		6.2		14.5		6.2

### Aim 1: ECE center changes and changes in child physical activity

Children in this sample were younger (38.9 ± 5.2 months) and a higher proportion were White (70.7%) compared to the 112 enrolled at baseline (41.1 ± 6.7 months, *p* = 0.02; 53.7% White, *p* = 0.001). No other demographic differences were present. Within the subset of 49 children, there was a significant increase in sedentary behavior by 3.6 ± 8.7 min/hour from baseline to follow-up, and total physical activity decreased by 3.6 ± 8.7 min/hour (*p* = 0.006 for both, Table 2). There was no change in MVPA between baseline and follow-up.

**Table 2:** Child PA recorded at the ECE center at Baseline and Follow-up^a^

	Baseline		Follow-up		Difference	*p*-value (*n* = 49)
	Mean ± SD		Mean ± SD		Mean ± SD	
	All (*n* = 112)	Subset (*n* = 49)	All (*n* = 83)^b^	Subset (*n* = 49)	(*n* = 49)	
SB minutes/hour	30.1 ± 6.0	29.9 ± 6.3	32.9 ± 7.0	33.5 ± 7.9	3.6 ± 8.7	0.006*
TPA minutes/hour	29.9 ± 6.0	30.1 ± 6.3	27.0 ± 7.0	26.5 ± 7.9	−3.6 ± 8.7	0.006*
MVPA minutes/hour	9.1 ± 3.8	9.0 ± 3.7	8.4 ± 3.7	8.1 ± 4.1	− 0.9 ± 4.4	0.17

*PA* Physical Activity, *SB* Sedentary Behavior, *TPA* Total Physical Activity, *MVPA* Moderate-to-Vigorous Physical Activity; Subset values are the data from children who completed measures both at baseline and follow-up (n = 49). ^a^*p*-value displayed is a result of a paired t-test of physical activity outcomes. ^b^83 participants includes all children with complete data at follow-up, which includes children from baseline and newly enrolled children at follow-up; *p < 0.05

Changes in ECE center practices, including active play and non-television screen-time, were associated with a change in child physical activity outcomes (Table 3). An increase in active play minutes was associated with child physical activity, including lower sedentary behavior and higher total physical activity (*p* = 0.02 for both). An increase in non-television screen-time, including tablets and handheld devices, was associated with higher sedentary behavior and lower total physical activity (*p* = 0.02) for both. Ten additional minutes of non-television screen-time was associated with 2.5 ± 1.0 additional minutes/hour of sedentary behavior. As for EPAO domains, higher revised-Sedentary Opportunities score, as in less non-television screen-time, was associated with an increase in MVPA (*p =* 0.02), and no other EPAO domains were associated with child physical activity.

**Table 3:** Association between ECE Center Changes and Change in Child Physical Activity at ECE center (*n* = 49 children)^a^

	Maximum Score	SB minutes/hour			TPA minutes/hour			MVPA minutes/hour		
		Beta	*SE*	*p*-value	Beta	*SE*	*p*-value	Beta	*SE*	*p*-value
*ECE Center Practice*										
Active Play (minutes)		−0.12	0.05	0.02*	0.12	0.05	0.02*	0.02	0.03	0.48
Television viewing (minutes)		0.21	0.28	0.46	−0.21	0.28	0.46	−0.08	0.10	0.39
Non-television screen-time (minutes)		0.25	0.10	0.02*	−0.25	0.10	0.02*	−0.10	0.04	0.06
Total Screen-time (minutes)		0.21	0.11	0.06	−0.21	0.11	0.06	−0.09	0.05	0.10
*ECE Center Environment*										
Active Opportunities	20	−1.09	0.71	0.13	1.09	0.71	0.13	0.22	0.52	0.67
Sedentary Opportunities	20	0.60	0.92	0.52	−0.60	0.92	0.52	−0.28	0.32	0.37
Sedentary Opportunities-Revised	20	−1.21	0.64	0.06	1.21	0.64	0.06	0.54	0.22	0.02*
Sedentary Environment	20	0.86	0.73	0.38	−0.86	0.73	0.38	0.50	0.40	0.21
Portable Play Environment	20	−0.56	0.83	0.50	0.56	0.83	0.50	0.02	0.28	0.93
Fixed Play Environment	20	−0.45	1.01	0.65	0.45	1.01	0.65	− 0.07	0.35	0.83
*ECE Center Staff Behavior*										
Staff Behaviors	20	0.08	0.96	0.93	−0.08	0.96	0.93	0.04	0.29	0.88
PA Training and Education	20	−0.01	0.81	0.98	0.01	0.81	0.98	−0.03	0.26	0.89
*ECE Center Policy*										
PA Policy^b^	20	N/A			N/A			N/A		
Total EPAO Score	160	−0.21	0.24	0.72	0.21	0.24	0.72	0.03	0.08	0.67

*SB* Sedentary Behavior, *TPA* Total Physical Activity, *MVPA* Moderate-to-Vigorous Physical Activity, *PA* Physical Activity, *EPAO* Environmental Policy Assessment and Observation; ^a^Assessed using mixed models account for baseline child sex, age, race, BMI percentile, and baseline score, and clustering within center. *p* < 0.05*; ^b^PA Policy is noted “N/A” as there were no changes in score from baseline to follow-up, and no change to evaluate

### Aim 2: ECE center changes between baseline and follow-up

About half of the ECE directors attended the roundtable discussion (*n* = 4) or viewed a recorded presentation (*n* = 4). Few participated in the webinar (*n* = 2), and only one attended all three events. Most ECE directors had some engagement (55%), and others had no engagement (22%) or high engagement (22%). The no engagement group had the highest baseline total EPAO score of the groups and showed minimal improvement (baseline: 83.6 ± 14.9, change: (− 0.8 ± 29.3), while the some engagement group had marginal improvement (baseline: 80.3 ± 17.1, change: 10.1 ± 7.4). The high engagement group had the lowest baseline total EPAO score and showed the most improvement of the groups (baseline: 49.1 ± 8.2, change: 27.8 ± 5.2). However, there was no significant difference in baseline, follow-up or change in total EPAO score between engagement groups (*p* > 0.05).

As shown in Table 4, there was a significant change in Active Opportunities and Staff Behaviors scores between baseline and follow-up (*p* < 0.05 for both). Total EPAO score improved for all centers by an average of 11.6 ± 15.6 points at follow-up. This change was not significant (*p* = 0.06), yet there was a moderate effect size (*d* = 0.72). The majority of screen-time viewed in the ECE centers consisted of non-television screen-time, which included handheld electronic devices, computers, and tablets. Television viewing was observed at baseline but not during the follow-up. Due to variability between centers this decrease from baseline was non-significant (*p* = 0.34). Specific to the policy, active play was observed for more than 60 min (including any teacher-led activity) in seven centers at baseline (77%) and in eight centers at follow-up (88%). At both time points, screen-time did not exceed 120 min when considering television, non-television, or total screen-time.

**Table 4:** ECE Center Practices, Environment, Staff Behavior, and Policies between Baseline and Follow-up measures (*n* = 9)^a^

	Maximum Score	Baseline Mean ± SD	Follow-up Mean ± SD	Difference Mean ± SD	*p*-value	Cohen’s *d*
*ECE Center Practice*						
Active Play (minutes)		88.0 ± 48.2	111.3 ± 42.7	23.3 ± 42.2	0.13	0.54
Television viewing (minutes)		2.8 ± 8.3	0.0 ± 0.0	−2.8 ± 8.3	0.34	0.33
Non-television screen-time (minutes)		26.7 ± 35.7	8.6 ± 17.8	− 18.1 ± 32.7	0.13	0.55
Total Screen-time (minutes)		29.4 ± 41.2	8.6 ± 17.8	−20.9 ± 39.2	0.14	0.52
*ECE Center Environment*						
Active Opportunities	20	11.4 ± 4.7	14.7 ± 3.6	3.3 ± 4.1	0.04*	0.79
Sedentary Opportunities	20	13.7 ± 2.6	13.3 ± 0.0	−0.4 ± 2.6	0.68	0.15
Sedentary Opportunities-Revised	20	10.7 ± 4.0	11.8 ± 2.9	1.1 ± 4.7	0.49	0.23
Sedentary Environment	20	5.9 ± 5.2	5.9 ± 2.2	0.0 ± 4.7	0.99	0.01
Portable Play Environment	20	9.8 ± 5.7	11.7 ± 4.6	1.9 ± 4.0	0.19	0.46
Fixed Play Environment	20	10.4 ± 3.2	9.4 ± 3.9	−1.0 ± 4.0	0.48	0.25
*ECE Center Staff Behavior*						
Staff Behaviors	20	12.2 ± 6.6	18.1 ± 2.9	5.9 ± 6.4	0.02*	0.92
PA Training and Education	20	4.7 *±* 3.2	4.2 ± 3.7	−0.5 ± 3.2	0.62	0.15
*ECE Center Policy*						
PA Policy	20	6.6 ± 7.1	6.6 ± 7.1	0.0 ± 0.0	0.99	*N/A*
Total EPAO Score	160	74.2 ± 20.1	85.7 ± 12.3	11.6 ± 15.6	0.06	0.72

*EPAO* Environmental Policy Assessment and Observation, *PA* Physical Activity; ^a^Values presented are mean and standard deviation; differences between baseline and follow-up assessed using a paired t-test. *p < 0.05. N/A

## Discussion

One year after a governmental policy change, ECE centers improved their active opportunities and staff behaviors related to physical activity. Unexpectedly, overall the children’s total physical activity decreased and sedentary behavior increased over the 1 year period. However, among those centers with improved physical activity practices (i.e. higher increases in observed active play time and decreased non-television screen-time), their children engaged in more total physical activity and less sedentary behavior. This study adds to the literature on observed changes in ECE centers and objective measurement of children’s physical activity. ECE environment and staff behavior improved after 1 year, and some children benefited specifically from these changes in ECE center practices at follow-up.

### ECE center changes and child physical activity

The ECE centers scored similarly on the EPAO components of Active Opportunities, Sedentary Opportunities, Staff Behaviors, Physical Activity Policy, as well as total EPAO score compared to the baseline measures of an intervention in Wisconsin ECE centers [22] and a cross-sectional study of Canadian ECE centers [7]. Compared to other studies of children’s physical activity in ECE centers, this sample engaged in slightly higher MVPA (8.9 min/hour) relative to other studies in 3–4 year old children during the school day that observed 7.0 min/hour [23] or 7.5–9.4 min/hour [24]. One reason for this may be that this sample wore the accelerometer for slightly longer on average (6.5 h) than other samples (5.5 h) [23, 24] allowing the children to accrue more physical activity including at the beginning and end of the school day, which may be more active periods with the available free play time. Changes in the ECE center practices (more active play and less non-television screen-time) were associated with more physical activity by the children. Interventions around active play in preschool children have been successful to decrease sedentary time and increase physical activity, as demonstrated in a randomized controlled trial of a family-focused active play intervention with 74 families of preschool age children [25].

Regarding screen-time, the use of non-television screen-time in the ECE center may be expected as a European cross-sectional study in young children (ages 0-to-5 years) observed about half of children begin using a non-television device, including a mobile device by 2 years of age [26]. Considering all devices, the recent 24-Hour Movement Guidelines for the Early Years from Canada and Australia stipulate children ages 3-to-4 years should not exceed more than 1 hour per day of screen-time [4, 27], in line with the American Academy of Pediatrics recommendation [28]. The Louisiana Department of Education licensing requirements allows for an additional hour of screen-time (2 hours total at the ECE center) compared to this 24-Hour Movement Guideline standard. Aside from time spent being sedentary; screen-time exposure may also contribute to obesity through exposure to high-calorie and non-nutritive foods and beverages, and reduce sleep duration [29]. Overall, a more cautious approach to screen-time, focusing on sparing amounts of educational screen-time, is encouraged [30].

Interestingly, the improvement in the revised-Sedentary Opportunities score, which represents a reduction in non-television screen-time, was related to more MVPA. Further, increased non-television screen-time (a specific sub-component of revised-Sedentary Opportunities) was associated with less total physical activity and more sedentary behavior. As the revised-Sedentary Opportunities score incorporates a metric of viewing non-television screen-time over 30 min; lessening prolonged periods of non-television screen-time may provide additional time to accumulate MVPA. Nonetheless, any reduction in non-television screen-time may be associated with any physical activity. There is limited investigation into non-television screen-time at the ECE center, as technology advances more quickly than research. As screen-based research measures evolve to meet the times, objective assessment of physical activity and child growth must continue alongside to better understand children’s usage of screen devices and how this may relate to their physical activity and sedentary time at ECE centers.

### ECE center practices, environment, staff behavior, and policies

Physical Activity Policy score did not change over the one-year period, unlike another study that evaluated government policy changes comparing 33 South Carolina ECE centers versus a comparator group of 26 North Carolina ECE centers, which observed a three point increase [31]. That study did find a significant increase in other EPAO scores (Fixed Environment, and Physical Activity Training and Environment) within the one-year period. The change in that study may be attributed to the other required aspects that were implemented as a part of the South Carolina state policy change, including providing a variety of play materials for indoor physical activity and requiring training for teachers on child physical activity [31]. The Louisiana Department of Education licensing requirements involved posting the state policy, providing more active play opportunities and limiting screen-time. In the current study, the observed changes in Active Opportunities and Staff Behaviors score may be a result of implementing the state policy (more physical activity opportunities and less screen-time viewing) or un-written policies and practices. Therefore, lack of change in handbook or posted specific written policies may not explain the lack of other changes within the ECE center.

A discussion of strengths and limitations is warranted. This study obtained both observed and objective measurement of behaviors within the ECE center using a validated observation tool (EPAO) and accelerometry. Moreover, this study captured information related to several types of screen-time (television and non-television) and may better inform state policies related to use of newer technology in the ECE centers. This study benefited from the opportunity to evaluate ECE centers and their children before and after implementation of statewide licensure changes. As the timing of some assessments did occur into the year 2017 due to delays in contracting, there is a chance that ECE centers changed their written policy after the regulations were first released in 2015–16 thus the baseline data collection was not a true baseline prior to policy implementation. However, this study did utilize handbooks for policy scoring and baseline policies were dated for the 2016–2017 school year at the beginning of the new licensing standards. Another limitation is that the ECE centers were aware of the day of observation and there is a potential the ECE center could have made changes before and during that day to present as a healthier environment. This study did not inform the ECE centers of the purpose of the study until after follow-up, all incentives given were school supplies not related to physical activity or screen-time, and surveys for directors also asked about nutrition-related practices and general licensure information not only physical activity or screen-time. This sample of Louisiana ECE centers may not be representative of the influence of state-level initiatives, as Louisiana ECE centers are in the lower cluster of state-licensure scales [8], and this sample was modest (9 ECE centers). Despite this small sample, significant findings were present in EPAO scores when assessing ECE center changes before and after the state policy. This study was unable to compare the ECE center changes with another state to provide a comparator or control condition for policy comparison, but did obtain engagement in licensure informational events for policy dissemination. Finally, the sample of children with repeated measures was modest (*n* = 49), which may be a reflection of design as the study evaluated the same classroom at both time points to retain the same teacher and physical classroom space. It is probable that as children aged, they were moved to another classroom and not able to participate in follow-up of the original classroom.

Future studies should focus on opportunities to improve ECE center policy, on the individual and state-level. In addition to requiring a minimum amount of time for teacher-led and free play as well as limits on screen-time, required training for staff in physical activity and sufficient fixed or portable play equipment in working order may also be beneficial for child physical activity. These two components improved after the implementation of specific state policy in South Carolina, and both can contribute to sustained physical activity in the centers [31]. Dissemination and implementation information is critical to address governmental changes and assess public health efforts. It is promising that most directors engaged in some state policy informational events, with the largest changes occurring in the high engagement group. Yet the high engagement group also had more room for improvement relative to other less engaged centers in this sample. Opportunities to engage all centers are needed for widespread change. Finally, investigation into the use of non-television screen-time and educational screen-time within the ECE center is warranted. Given the spectrum of views on the purpose or usefulness of screen-time in young children [32], opportunities to meet expectations of ECE teachers for providing children with both physical activity and educational opportunities are needed.

In summary, this natural experiment assessing ECE center changes and child physical activity found there were improvements in ECE center setting, and specific ECE center practices led to increases in child physical activity and concomitant reductions in sedentary time. ECE centers provided more opportunities for children to be physically active and staff behaviors that facilitated physical activity were more frequent. Further, there was an association between changes in ECE centers’ practices with change in children’s behavior: provision of more time in active play and less time in screen viewing were related to an increase in children’s total physical activity. As changes within the ECE center resulted in beneficial child movement, these results provide further evidence that ECE center policy and practice is an important place for child health and focus for public health practitioners. Encouraging state policies that facilitate physical activity through the ECE setting and staff training may provide additional opportunities for children to be physically active and less sedentary in ECE centers.

## Supplementary information

**Additional file 1: Supplementary Table S1.** STROBE Statement.

## Data Availability

The datasets used and/or analyzed during the current study are available from the corresponding author on reasonable request.

## Abbreviations

ANOVA: Analysis of Variance
BMI: Body Mass Index
ECE: Early Childhood Education
EPAO: Environmental Policy Assessment and Observation
MVPA: Moderate-to-Vigorous Physical Activity
PA: Physical Activity
TPA: Total Physical Activity
TV: Television
